# Adrenal Ganglioneuroma: A Rare Tumor of the Autonomic Nervous System

**DOI:** 10.7759/cureus.12398

**Published:** 2020-12-31

**Authors:** Mark A Burroughs, Ivan Urits, Omar Viswanath, Alan D Kaye, Jamal Hasoon

**Affiliations:** 1 General Surgery, Methodist Dallas Medical Center, Dallas, USA; 2 Anesthesia, Critical Care and Pain Medicine, Beth Israel Deaconess Medical Center, Harvard Medical School, Boston, USA; 3 Pain Management, Valley Pain Consultants - Envision Physician Services, Phoenix, USA; 4 Anesthesiology, Louisiana State University Health Shreveport, Shreveport, USA

**Keywords:** ganglioneuroma, surgery

## Abstract

Ganglioneuromas (GNs) are benign, differentiated tumors that are derived from neural crest sympathogonia. They can be found anywhere in the body along autonomic ganglia. These tumors are seen most often in individuals over 10 years of age and are usually asymptomatic. However, GNs can grow large and cause symptoms due to mass effect. GNs are rarely located within the suprarenal gland and are benign tumors that are often diagnosed late in their course due to mass effect or found incidentally on imaging studies. We describe a case of a 22-year-old female who presented to clinic with vague abdominal pain. She underwent an extensive workup which ultimately revealed a rare presentation of a left suprarenal GN that required surgical removal. She underwent an extensive workup which ultimately revealed a left suprarenal GN that required surgical removal. The patient underwent laparotomy and surgical excision of the mass and made a full recovery.

## Introduction

Ganglioneuromas (GNs) are benign, differentiated tumors that are derived from neural crest sympathogonia. These tumors can be found anywhere in the body along autonomic ganglia. However, the two most common locations are the retroperitoneum (32%-52% of cases) and posterior mediastinum (39%-43%), followed by the cervical region (8%-9%) [1]. In a series of 46 patients with abdominal GNs, the tumor was located in the extra-adrenal retroperitoneum in 27 patients (59%) and in the adrenal gland in 19 patients (41%) [1]. These tumors are seen most often in individuals over 10 years of age and are usually asymptomatic. However, GNs can grow large enough to cause symptoms due to mass effect. We describe a case of a 22-year-old female who presented to clinic with vague abdominal pain that did not respond to conservative treatment. 

## Case presentation

A 22-year-old female with no significant past medical history presented to clinic for evaluation of vague abdominal pain. She reported a history of progressively worsening pain in the epigastric region along with nausea and bloating that had been ongoing for the past two months. She had been followed by her primary care physician and reported minimal response to over-the-counter analgesics including acetaminophen, nonsteroidal anti-inflammatory drugs, and antacids. She also noted no improvement with diet changes. Initial bloodwork revealed elevated liver function tests on workup and she was referred for imaging evaluation.

**Figure 1:**
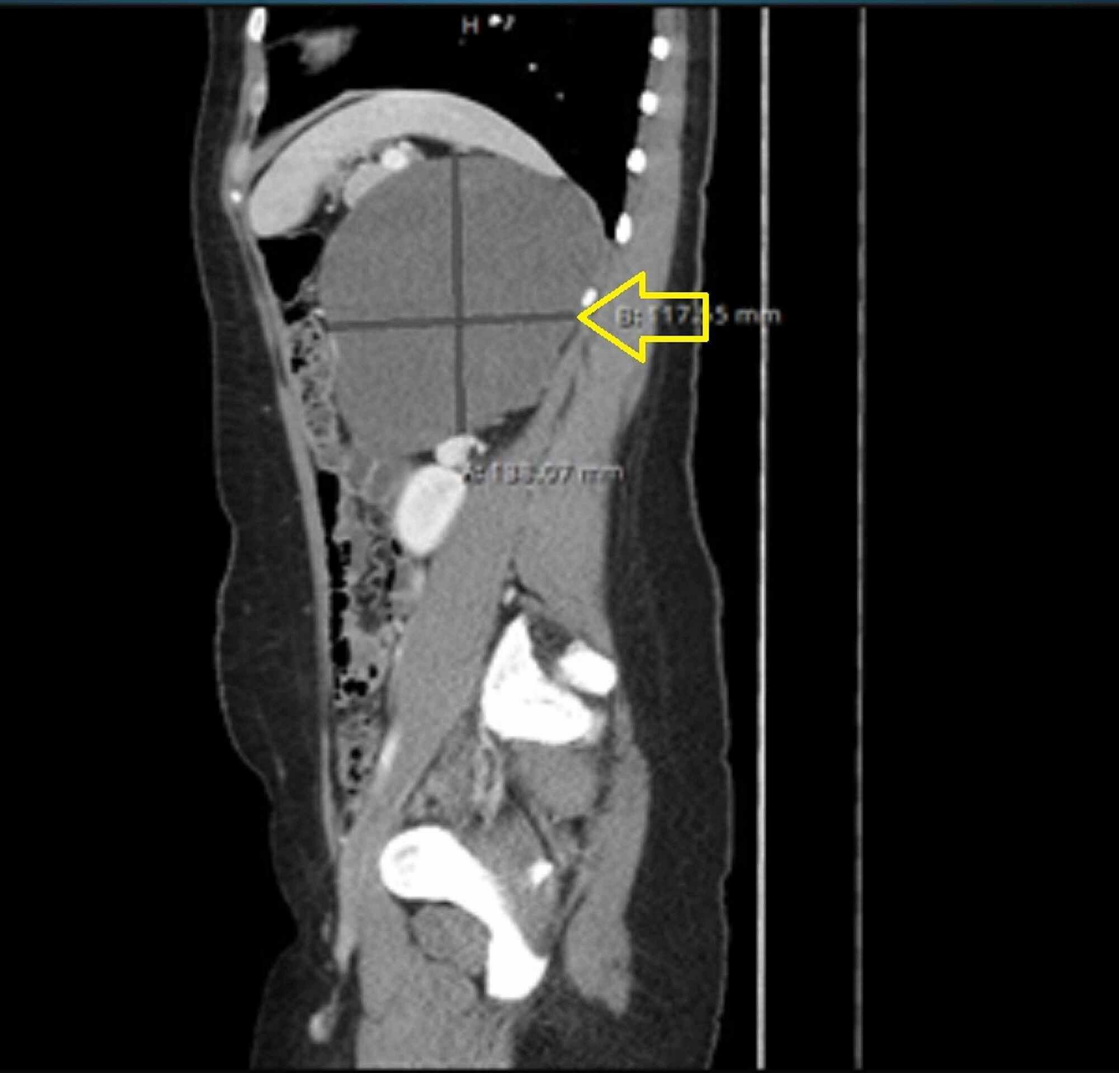
CT abdomen/pelvis sagittal view. The yellow arrow demonstrates a large suprarenal mass causing displacement of adjacent structures.

**Figure 2:**
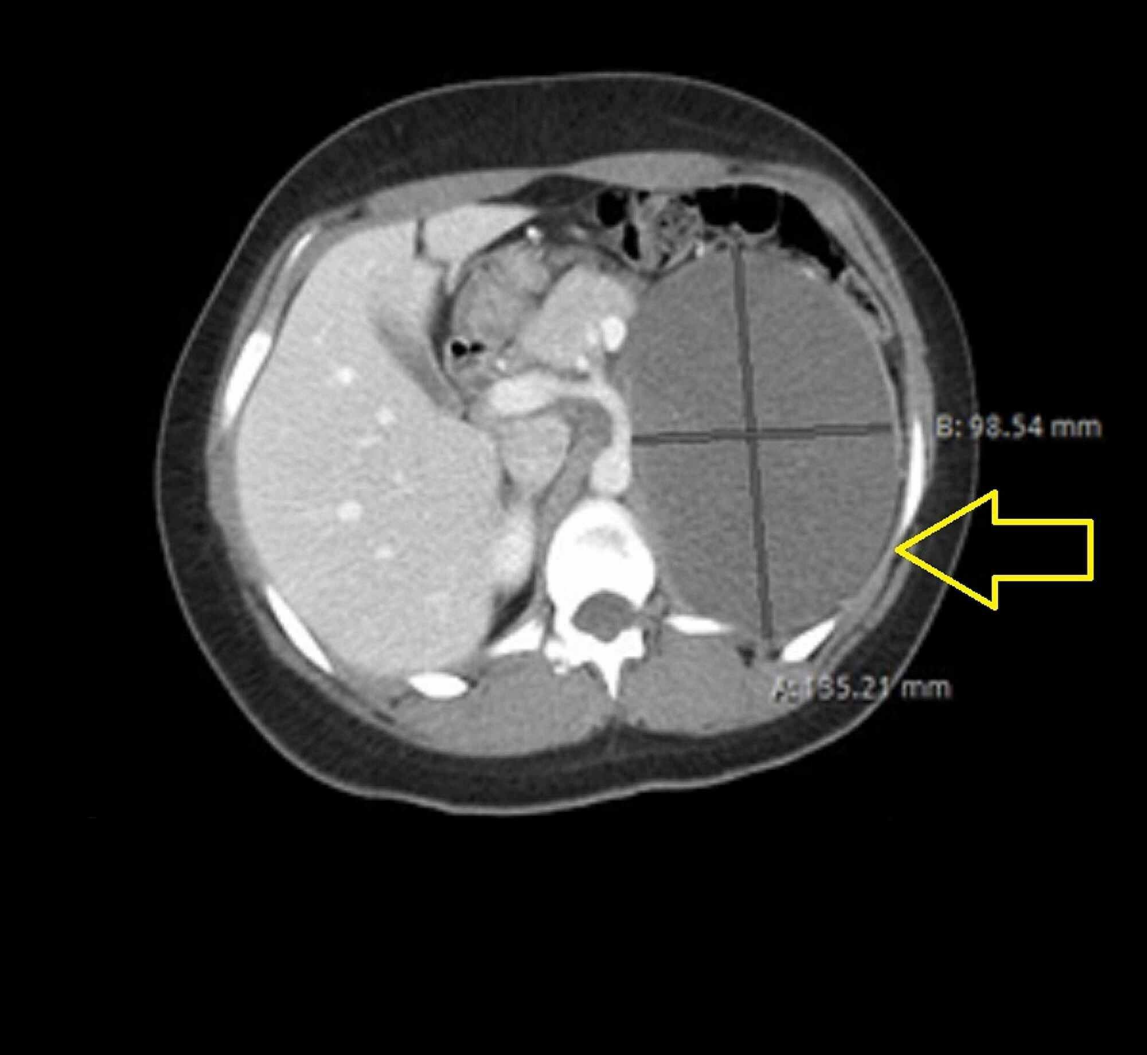
CT abdomen/pelvis axial view. The yellow arrow demonstrates a large mass causing displacement of adjacent structures.

An ultrasound was performed which demonstrated cholelithiasis without cholecystitis and a cystic structure between the left kidney and the spleen with atypical features. The patient was sent for a subsequent CT of the abdomen and pelvis with and without contrast which revealed a large left upper quadrant cystic mass with septations and calcifications. The mass compressed multiple structures and displaced the pancreas anteriorly and the spleen laterally. An MRI was performed and demonstrated a 10.0 cm x 12.2 cm x 13.3 cm [anterioposterior (AP), transverse, and craniocaudal] retroperitoneal mass with predominate fat signal. Displacement of the left kidney inferiorly, spleen laterally, and stomach superiorly led to a presumed suprarenal origin (Figures 1-2). 

The patient underwent laparotomy and surgical excision of the mass. Histopathological examination after open surgical excision demonstrated a left suprarenal GN negative for malignancy. The patient had an uneventful postoperative course. Her postoperative pain was well managed with patient controlled analgesia (PCA) utilizing low dose hydromorphone. She had no complications and made a full recovery.

## Discussion

Ganglioneuromas are benign differentiated tumors derived from neural crest sympathogonia. These tumors occur mainly in adolescents and young adults. The incidence of GNs is approximated to be one per million people [2]. They can be found anywhere in the body along the autonomic ganglia. However, the two most common locations are the retroperitoneum (32%-52% of cases) and posterior mediastinum (39%-43%), followed by the cervical region (8%-9%) [1]. In a series of 46 patients with abdominal GN, the tumor was located in the extra-adrenal retroperitoneum in 27 patients (59%) and in the adrenal gland in 19 patients (41%) [1].

Ganglioneuromas are seen most often in individuals over 10 years of age and are usually asymptomatic. They are commonly discovered incidentally on imaging. However, GNs can grow large enough to cause symptoms due to mass effect as was demonstrated in our patient. Although most are nonfunctioning, approximately 37% of GNs secrete catecholamines and their metabolites. These secretions can include vasoactive intestinal peptide which can lead to profuse watery diarrhea in patients [3].

A diagnosis of GN should be suspected in patients with adrenal tumors that demonstrate: (1) no hormonal hypersecretion, (2) presence of punctate or discrete calcifications, (3) absence of vessel involvement, and (4) a low nonenhanced T1-weighted signal with a late and gradual enhancement on dynamic MRI [4].

Differential diagnoses of GNs include: ganglioneuroblastoma, neuroblastoma, composite pheochromocytoma, adrenal cortical adenoma, and adrenocortical carcinoma [5]. Confirmation of the diagnosis requires histopathological examination and analysis. Microscopically, GNs are composed of mature Schwann cells, ganglion cells, and perineural cells within a fibrous stroma [6]. Due to the availability and high sensitivity of ultrasound, CT and MRI imaging modalities, GNs should be included in differential diagnosis for asymptomatic masses along autonomically innervated tissues [3]. Biopsy with histological examination is the current diagnostic gold standard and should be thorough to exclude ganglioneuroblastoma or neuroblastoma foci, signifying a poorer prognosis [7]. Definitive treatment of GNs is either laparoscopic or open surgical resection of the tumor. Laparoscopic resection is recommended for tumors less than 6 cm [6]. Prognosis in these patients is excellent after resection.

Furthermore, while a great majority of GNs are sporadic, familial disposition as well as associations with Turner Syndrome and multiple endocrine neoplasm II (MENII) have been reported [4]. Our patient had no family history or associated risk factors for a GN.

## Conclusions

Ganglioneuromas are rarely located within the suprarenal gland and are benign tumors that are often diagnosed late in their course due to mass effect, or found incidentally on imaging studies. We have described a case of a 22-year-old female who was diagnosed with this rare tumor after vague abdominal symptoms prompted further imaging studies. Prognosis in these patients is excellent after resection.

## References

[1] Rha SE, Byun JY, Jung SE, Chun HJ, Lee HG, Lee JM (2003). Neurogenic tumors in the abdomen: tumor types and imaging characteristics. Radiographics.

[2] Dąbrowska-Thing A, Rogowski W, Pacho R, Nawrocka-Laskus E, Nitek Ż (2017). Retroperitoneal ganglioneuroma mimicking a kidney tumor. A case report. Pol J Radiol.

[3] Leão RR, Pereira BJ, Borges R, Grenha V (2013). Adrenal ganglioneuroma: a rare incidental finding. BMJ Case Rep.

[4] Allende DS, Hansel DE, MacLennan GT (2009). Ganglioneuroma of the adrenal gland. J Urol.

[5] Mylonas KS, Schizas D, Economopoulos KP (2017). Adrenal ganglioneuroma: what you need to know. World J Clin Cases.

[6] Chen P, Lin C, Jin L (2017). A case report of giant adrenal ganglioneuroma. Urol Case Rep.

[7] Gupta R, Dinda AK (2007). Ganglioneuroma of the adrenal gland: a rare case. Indian J Pathol Microbiol.

